# Diagnostic practices and estimated burden of tuberculosis among children admitted to 13 government hospitals in Kenya: An analysis of two years’ routine clinical data

**DOI:** 10.1371/journal.pone.0221145

**Published:** 2019-09-04

**Authors:** Jacquie Narotso Oliwa, David Gathara, Morris Ogero, Michaël Boele van Hensbroek, Mike English, Anja van’t Hoog, the Clinical Information Network

**Affiliations:** 1 KEMRI-Wellcome Trust Research Programme, Health Services Research Group, Nairobi, Kenya; 2 University of Nairobi, Department of Paediatrics and Child Health, Nairobi, Kenya; 3 Amsterdam Institute for Global Health and Development, Amsterdam, The Netherlands; 4 The Academic Medical Centre, University of Amsterdam, Department of Global Health, Amsterdam, The Netherlands; 5 Oxford University, Nuffield Department of Medicine, Oxford, England, United Kingdom; The University of Hong Kong, CHINA

## Abstract

**Background:**

True burden of tuberculosis (TB) in children is unknown. Hospitalised children are low-hanging fruit for TB case detection as they are within the system. We aimed to explore the process of recognition and investigation for childhood TB using a guideline-linked cascade of care.

**Methods:**

This was an observational study of 42,107 children admitted to 13 county hospitals in Kenya from 01Nov 15-31Oct 16, and 01Nov 17-31Oct 18. We estimated those that met each step of the cascade, those with an apparent (or “Working”) TB diagnosis and modelled associations with TB tests amongst guideline-eligible children.

**Results:**

23,741/42,107 (56.4%) met step 1 of the cascade (≥2 signs and symptoms suggestive of TB). Step 2(further screening of history of TB contact/full respiratory exam) was documented in 14,873/23,741 (62.6%) who met Step 1. Step 3(chest x-ray or Mantoux test) was requested in 2,451/14,873 (16.5%) who met Step 2. Step 4(≥1 bacteriological test) was requested in 392/2,451 (15.9%) who met Step 3. Step 5(“Working TB” diagnosis) was documented in 175/392 (44.6%) who met Step 4. Factors associated with request of TB tests in patients who met Step 1 included: i) older children [AOR 1.19(CI 1.09–1.31)]; ii) co-morbidities of HIV, malnutrition or pneumonia [AOR 3.81(CI 3.05–4.75), 2.98(CI 2.69–3.31) and 2.98(CI 2.60–3.40) respectively]; iii) sicker children, readmitted/referred [AOR 1.24(CI 1.08–1.42) and 1.15(CI 1.04–1.28) respectively]. “Working TB” diagnosis was made in 2.9%(1,202/42,107) of all admissions and 0.2%(89/42,107) were bacteriologically-confirmed.

**Conclusions:**

More than half of all paediatric admissions had symptoms associated with TB and nearly two-thirds had more specific history documented. Only a few amongst them got TB tests requested. TB was diagnosed in 2.9% of all admissions but most were inadequately investigated. Major challenges remain in identifying and investigating TB in children in hospitals with access to Xpert MTB/RIF and a review is needed of existing guidelines.

## Introduction

The true burden of TB in children is unknown, but modelling estimates suggest it could be a leading cause of death in children, a “hidden epidemic”, with up to 65% of paediatric TB cases potentially missed each year [1–3]. Difficulties in accurately identifying cases of TB in children and lack of good surveillance data have made it challenging to quantify the actual burden of childhood TB [4]. According to World Health Organisation (WHO) estimates, there are 21,000 new childhood TB cases in Kenya, but only 7,648 (36%) are notified [5, 6]. The recent Kenya TB prevalence survey with participants >15yrs revealed 75% of TB cases had presented to health facilities with suggestive symptoms but were never diagnosed [7]. The proportion of younger children who present to health facilities and go undiagnosed in Kenya is presently unknown, but is presumed to be as high [6, 8, 9]. Data on burden of TB amongst paediatric admissions and clinicians’ diagnostic practices in resource-limited settings are also sparse, and where they exist, they mostly present data from single tertiary hospitals in better-resourced settings [10–12]. However, where TB is prevalent in the population one might expect to see TB more commonly amongst admissions.

In efforts to improve TB case detection and treatment, the WHO develops guidelines that national TB programmes adapt [13, 14]. According to Kenyan TB guidelines, diagnosing TB in children relies on careful history and physical examination to identify suggestive signs and symptoms of cough, fever, lethargy and growth faltering, followed by investigations including chest x-ray, tuberculin skin test (Mantoux), Xpert MTB/RIF and/or culture [13]. Unfortunately, TB diagnosis in children is complicated by low sensitivity and specificity of symptoms and lack of appropriate point-of-care diagnostic tests [15, 16]. Guidelines provide clinical decision support, therefore assessing how well health workers adhere to these guidelines provides opportunity to evaluate quality of TB care. Guidelines suggest clinicians should follow a series of steps spanning assessment, diagnosis and treatment, which can be represented in a care cascade. Cascades have been extensively used in HIV studies to describe quality of care [17–21], and are now increasingly used in TB care [22–25]. We identified one study using the cascade concept in paediatric TB [26] and another in adolescent TB [27] but none specifically looking at paediatric in-patient care.

**Fig 1:**
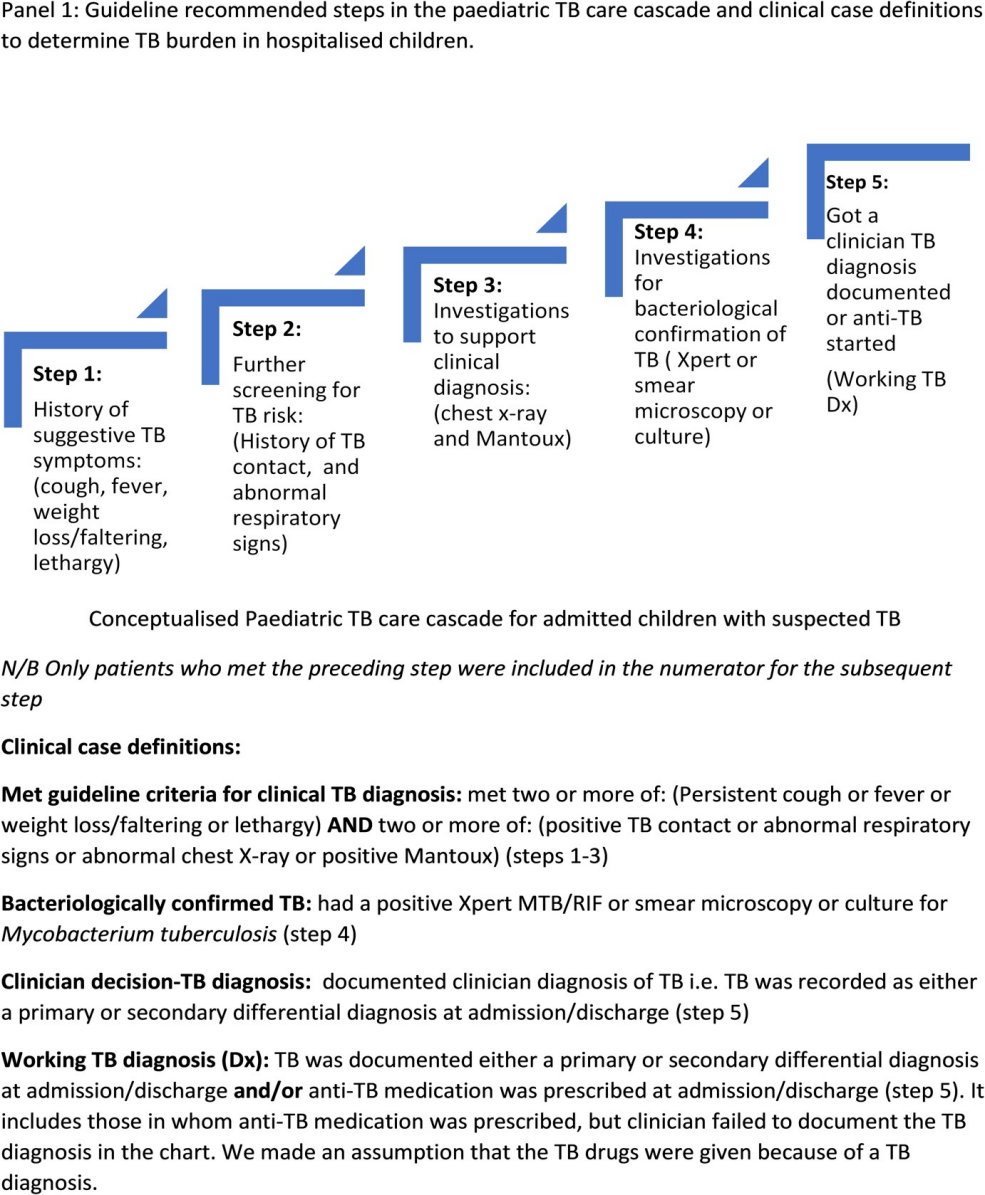
Panel showing guideline-recommended diagnostic steps and clinical case-definitions of TB in the study.

This paper explored the process of recognition and investigation for childhood TB using a large longitudinal observational data set of hospitalised children in Kenya, a high burden TB country. Specifically, it aimed to: i) describe a guideline-linked paediatric TB care cascade (Fig 1) to audit clinician TB diagnostic practices; ii) to explore associations with use of TB diagnostic tests among children who entered the cascade; and iii) to estimate burden of TB diagnosis made in children admitted to Kenyan hospitals using various case-definitions. Findings may help explain a major gap in the cascade of paediatric TB care in Kenya. This understanding could help develop targeted strategies to improve paediatric TB case detection and care.

## Methods

### Study design and setting

We report findings from an observational study using data from the Clinical Information Network (CIN), a partnership established in 2013 between Kenya Medical Research Institute (KEMRI), Ministry of Health (MoH) Kenya, Kenya Paediatric Association (KPA) and 13 public county referral hospitals [28, 29]. The network collects standardized routine data on paediatric admissions with the aim of promoting adoption of evidence-based interventions and improving quality of care. Data are abstracted from a standard paediatric admission record (PAR) form (see S1 Chart) linked to national clinical guidelines defining key symptoms, signs, investigations, diagnostic formulations and treatment plans for the most common childhood illnesses [30]. The data are then used to provide feedback every 2–3 months on patient process of care and outcomes to participating CIN sites to guide quality improvement activities. Over time, these audit and feedback processes have helped improve documentation practices and guideline adoption for common childhood illness like pneumonia, malnutrition and diarrhoea [31–33]. A detailed description of the CIN and data management processes have been documented in earlier publications [28, 29].

The data available within this network provided an opportunity to do a clinical audit of the extent to which the Kenya paediatric TB diagnostic guidelines are followed for children admitted to typical government county referral hospitals. All sites had access to an Xpert MTB/RIF machine and smear microscopy for bacteriological tests, all had X-ray facilities, but reagents for Mantoux testing were sporadically available nationwide throughout, and there was a shortage of Xpert MTB/RIF cartridges for some months in 2018. None of the study hospitals have been receiving feedback specific to TB from CIN. Table 1 summarises CIN study hospitals’ characteristics that we used in the exploration of hospital level characteristics and how they may influence case detection of TB in children.

**Table 1:** Hospital and respective county characteristics.

Hospital	Paediatric Wards Bed Capacity	County level parameters					
		Xpert MTB/RIF sites /county	TB CNR /100,000 ^a^	Proportion of TB cases among <15yr ^b^ (%)	Proportion of HIV cases ^c^ (%)	Proportion of Stunting in <5yr ^d^ (%)	Malaria Transmission rates
**H1**	32	4	219	10	6.7	22	Moderate-high
**H2**	29	2	265	13	3.3	27	Very low-low
**H3**	35	4	120	10	4.0	28	Moderate-high
**H4**	24	2	167	11	3.4	15	Very low-low
**H5**	63	2	227	13	3.1	17	Very low-low
**H6**	67	5	236	7	5.6	16	Very low-low
**H7**	29	8	214	6	19.9	18	Moderate-high
**H8**	38	1	120	9	5.2	29	Moderate-high
**H9**	35	3	205	5	4.5	27	Very low-low
**H10**	41	15	304	7	6.1	17	Very low-low
**H11**	42	15	304	7	6.1	17	Very low-low
**H12**	32	2	167	11	3.4	15	Very low-low
**H13**	21	2	131	7	4.7	24	Moderate-high

^a^ County TB Case Notification Rates (CNR) from 2017 National TB Programme Annual Report [6]

^b^ From 2017 National TB Programme Annual Report [6]

^c^ County HIV prevalence/proportions from National AIDS and STI Control County estimates for 2016 [34]

^d^ County Malnutrition levels from KDHS 2014 [35]

### Study participants, study size and data sources

**Fig 2:**
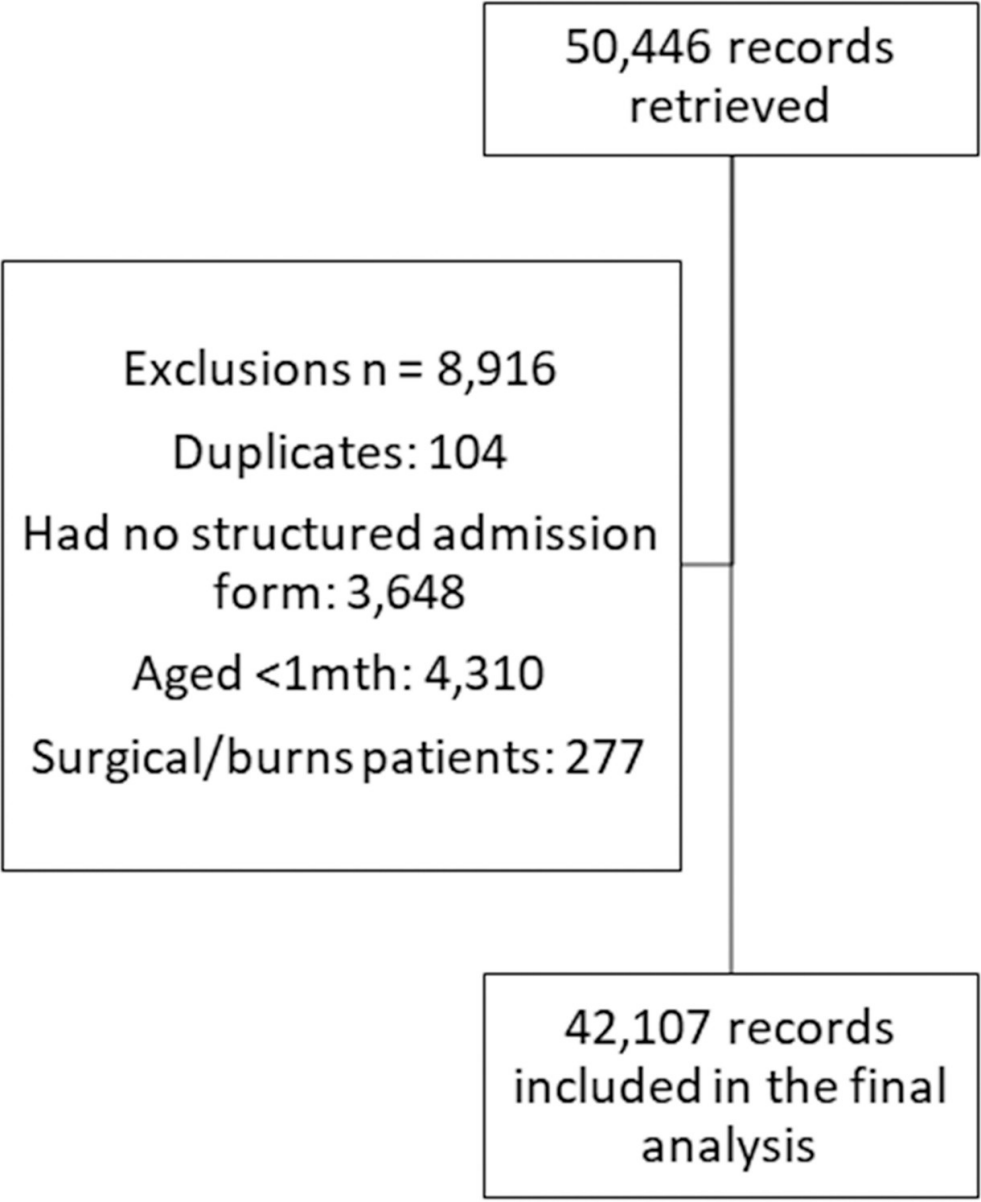
Flowchart of inclusion and exclusion criteria.

We included routine hospital data for all paediatric admissions to CIN hospitals between from 1^st^Nov 2015-31^st^ Oct 2016, and from 1^st^ Nov 2017-31^st^ Oct 2018 (24 calendar months), which gave us a total of 50,466 records. We excluded Nov 2016-Oct 2017 because Kenya went through prolonged health worker strikes, which adversely affected admissions to public hospitals [36]. We opted to use data for one calendar year prior to and after strikes when the health care system was relatively stable. We also excluded: i) surgical and burns patients, as they had little to no data on process of care; ii) those who were less than a month old, as tuberculosis in the neonatal age manifests differently and uses a different diagnostic approach; and iii) patients whose admission was not recorded in structured record forms (see flowchart Fig 2). The Kenya Medical Research Institute (KEMRI) Scientific and Ethical Review Committee (SSC Number 2465) approved the CIN study enabling use of de-identified data without individual patient consent.

### Study variables and statistical methods

To describe diagnostic practices and explore associations with use of TB diagnostic tests, we used the Kenya National paediatric TB guidelines (S1 Fig) [13], to develop variables to demonstrate documentation of signs and symptoms suggestive of TB and use of investigations conceptualised in a cascade i.e. where only those who met the preceding step were included in the numerator for the next step (Fig 1 and S1 Table). We also described cases who partially met criteria for each step by missing one or more of the preceding steps.

The main outcome for exploratory modelling was documentation of request for any one of the TB diagnostic tests (chest X-ray, Mantoux test, smear microscopy, Xpert MTB/RIF or culture) in those admitted with two or more signs and symptoms of TB (step 1 of the cascade), chosen pragmatically because the guidelines are unclear as to when investigations should be done and for which category of patients. Explanatory variables chosen *a priori* in models exploring use of tests included: child’s age; gender; presence of a danger sign of severe illness; whether they were a readmission or referral case; and whether they had co-morbidities of HIV, malnutrition or pneumonia. Hospital level factors included whether patients came from facilities that were: i) busy (high admissions)- mean number of admissions was 3,239, so those admitting more were considered busy and included H1, H3, H6, H8, H10, H12; ii) low or high malaria transmission sites (high transmission sites were H1, H3, H7, H8, H13); iii) and whether they were from high HIV prevalence counties i.e. above the group average of 5.9%, and included H1, H7, H10, H11. We also divided data into time periods (monthly, quarterly, bi-annually and annually) to explore effect of time on use of TB tests in study hospitals. S1 Table shows definitions of variables of interest.

To estimate the burden of TB amongst admissions to paediatric wards, we developed case definitions for clinician TB diagnosis, “Working TB” diagnosis and those who met guideline-recommended criteria for clinical TB or bacteriologically-confirmed TB (see panel in Fig 1 and S1 Fig).

We present summary statistics including frequencies, proportions, means, medians and ranges for categorical and continuous variables as appropriate for descriptive analysis. Variables of interest were used in both univariate and multivariate hierarchical models to explore possible factors that could explain the greatest identified gap in the cascade of TB care (use of TB investigations in children who entered the cascade) with hospital as a random effect. Backward selection was used to build the multivariate model, iteratively removing the least significant predictors at a time. We did not impute missing data as missingness of clinical information is itself an aspect of the cascade itself, which we needed to note, as part of the audit of clinical care.

Likelihood ratio tests and quantile-quantile plots of residuals were then used to determine best fit. Odds ratios were reported with 95% confidence intervals for explanatory variables, exploring for interactions in pre-specified covariates to determine effect modification. The final adjusted model converged at five integration points using a complete case analysis.

## Results

Table 2 shows patient characteristics from the two-time periods pre- and post-strikes separately and pooled, with the last column showing data not documented/information missing from patients’ files as part of the clinical audit. The footnote in Table 2 and S1 Table shows definitions of variables. We ended up with 42,017 patients pooled from the two-time periods. Median age was 19 months (IQR 9, 47 mth) and 55.3% of all these admissions (N = 42,107) were male. Fever was the most common presenting complaint followed by cough in 66.6% (28,032/42,107) and 50.8% (21,408/42,107) respectively. Nearly a third (12,485/42,107) were reported to have growth faltering, while 17.2% (7,400/42,107) had lethargy-defined by AVPU scale < Alert OR not able to drink. 30% (12,485/42,107) had a danger sign while nearly half (19,849/42,107) had an abnormal respiratory sign. Most had an acute illness presentation with median pre-admission history of illness being three days (IQR 1, 5 days). Approximately 10% (4,234/42,107) were readmissions and 16% (6,846/42,107) were referrals from lower-level facilities. Nearly 2% (778/42,107) were either HIV infected or sero-exposed; while 19,018/42,107 (45.2%) had an admission diagnosis of pneumonia or respiratory tract infection. Median stay in hospital was three days (IQR 2, 6 days). 2,448 (5.8%) of patients died during their hospital stay.

**Table 2:** Descriptive characteristics of patients admitted to CIN hospitals in the study period.

Characteristic	Period 1 ^a^ Admissions N = 23,194	Period 2 ^b^ Admissions N = 18,913	Pooled Data All admissions N = 42,107	Not documented /missing information (from pooled data)
**Age in months: Median (IQR)**	20 (9, 48)	18 (9, 43)	19 (9, 47)	433 (1.0)
**Male**	12,683 (54.7)	10,593 (56.0)	23,274 (55.3)	161 (0.4)
**Any cough**	11,586 (50.0)	9,822 (51.9)	21,408 (50.8)	3,789 (9.0)
**Cough >2weeks**	756 (3.3)	836 (4.4)	1,592 (3.8)	21,078 (50.1)
**Fever**	15,522 (66.9)	12,510 (66.1)	28,032 (66.6)	3,916 (9.3)
**History of TB contact**	228 (1.0)	320 (1.7)	548 (1.3)	11,546 (27.4)
**Underweight (-2SD WAZ)**	5,809 (25.0)	4,927 (26.1)	10,736 (25.5)	4,629 (11.0)
**Growth Faltering ^c^**	6,859 (29.6)	5,990 (31.7)	12,849 (30.5)	-
**Lethargic ^d^**	4,143 (17.9)	3,256 (17.2)	7,400 (17.6)	3,790 (9.0)
**Any danger sign (severe disease) ^e^**	6,653 (28.7)	5,831 (30.8)	12,485 (30.0)	3,444 (8.2)
**Any abnormal respiratory sign ^f^**	10,763 (46.4)	9,086 (48.0)	19,849 (47.1)	3,442 (8.2)
**Length of illness (median, IQR)**	3 (1, 5)	3 (1, 5)	3 (1, 5)	5,133 (12.2)
**Readmission**	2,644 (11.4)	1,590 (8.4)	4,234 (10.1)	7,244 (17.2)
**Referral**	4,015 (17.3)	2,831 (15.0)	6,846 (16.3)	7,201 (17.2)
**HIV infected/exposed**	488 (2.1)	290 (1.5)	778 (1.9)	-
**Pneumonia/RTI**	10,382 (44.8)	8,637 (45.7)	19,018 (45.2)	-
**Length of stay-days (median, IQR)**	3 (2, 6)	4 (2, 7)	3 (2, 6)	32 (0.08)
**Died**	1,302 (5.6)	1,146 (6.1)	2,448 (5.8)	3 (0.01)

^a^ Period 1: 1^st^ November 2015 to 31st October 2016

^b^ Period 2: 1st November 2017 to 31st October 2018

^c^ Growth Faltering: Either WAZ <-2SD OR admission diagnosis of malnutrition/failure to thrive OR had a prescription for supplementary feeds

^d^ Lethargic: AVPU < Alert OR not able to drink

^e^ Any danger sign: Central cyanosis OR AVPU < Alert OR not able to drink OR grunting OR received oxygen

^f^ Any abnormal respiratory sign: High respiratory rate (for age) OR received oxygen OR central cyanosis OR indrawing OR grunting OR acidotic breathing OR crackles OR wheeze

### Paediatric TB diagnostic practices

**Fig 3:**
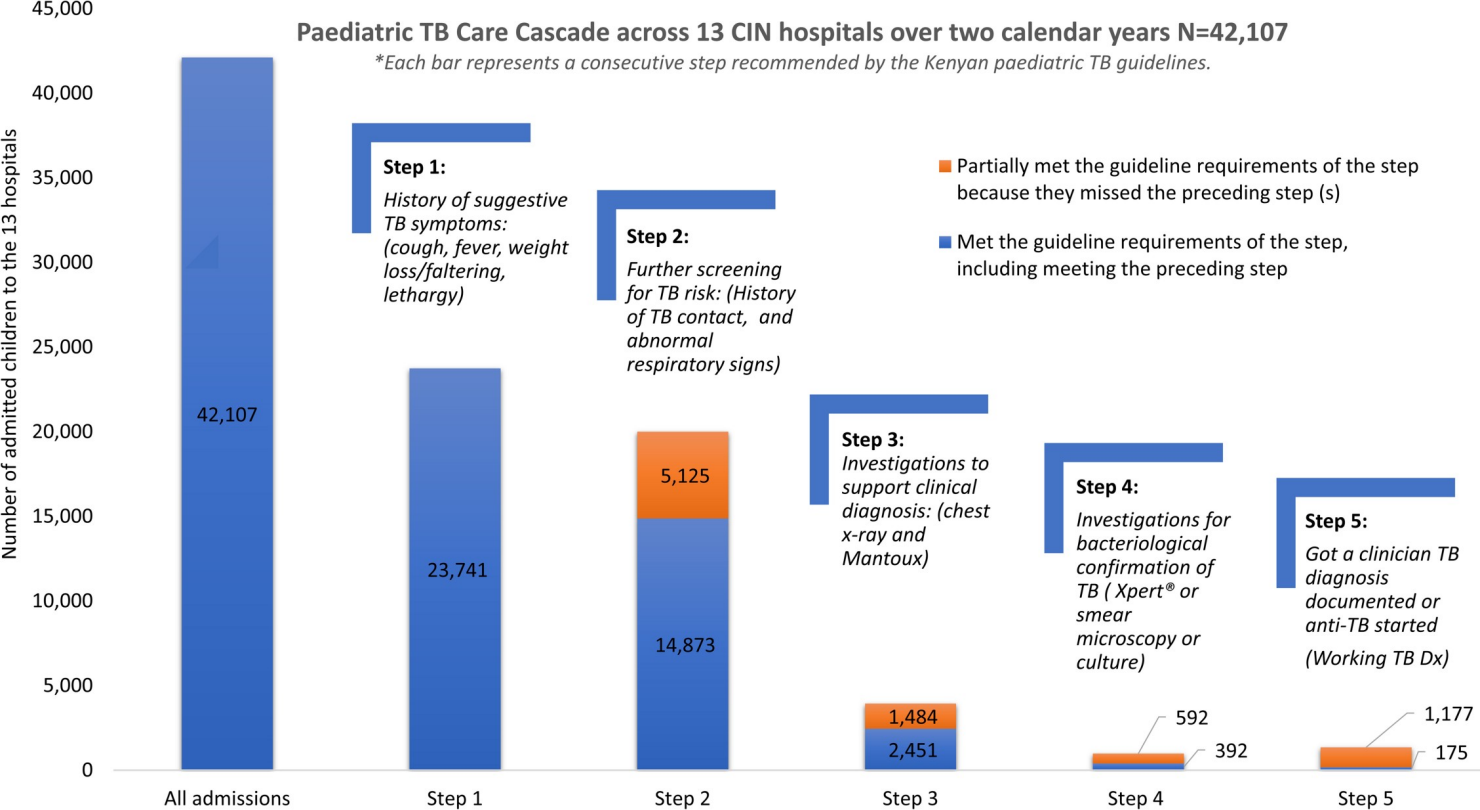
Paediatric TB Care Cascade across the 13 hospitals.

Using the conceptualised paediatric TB care cascade (panel Fig 1) to examine diagnostic practices amongst paediatric admissions with results in Fig 3 (with blue bars illustrating those who met the criteria for each step, and the orange bars illustrating those who only partially met criteria, because they missed one or more of the preceding cascade steps). 23,741/42,107 (56.4%) of all admissions met step 1 of the cascade, with two or more signs and symptoms suggestive of TB (i.e. cough, fever, weight loss/growth faltering, lethargy). Step 2, further screening by documenting history of TB contact or full respiratory exam was done in 14,873/23,741 (62.6%) of those who met Step 1 (Fig 3 blue bar). An additional 5,125 patients were assessed in line with Step 2 guidance but had not met criteria for Step 1 (Fig 3 orange bar). Step 3, having a chest x-ray or Mantoux test was met in 2,451 (16.5%) of the 14,873 who met Step 2 (Fig 3 blue bar), while 1,484 only partially met Step 3 (Fig 3 orange bar). Step 4 of having at least one bacteriological test done was noted in 392 (15.9%) of the 2,451 who met Step 3 (Fig 3 blue bar); while an additional 592 patients only partially met Step 4 (Fig 3 orange bar). In Step 5, getting a Working TB diagnosis was observed in 175 (44.6%) of the 392 patients who met Step 4 (Fig 3 blue bar). There were an additional 1,177 patients who got a Working TB diagnosis, but missed one or more of the preceding cascade steps (Fig 3 orange bar). S2 Table and S2 Fig show the absolute numbers and full patient flow chart of the clinician decision making process.

**Fig 4:**
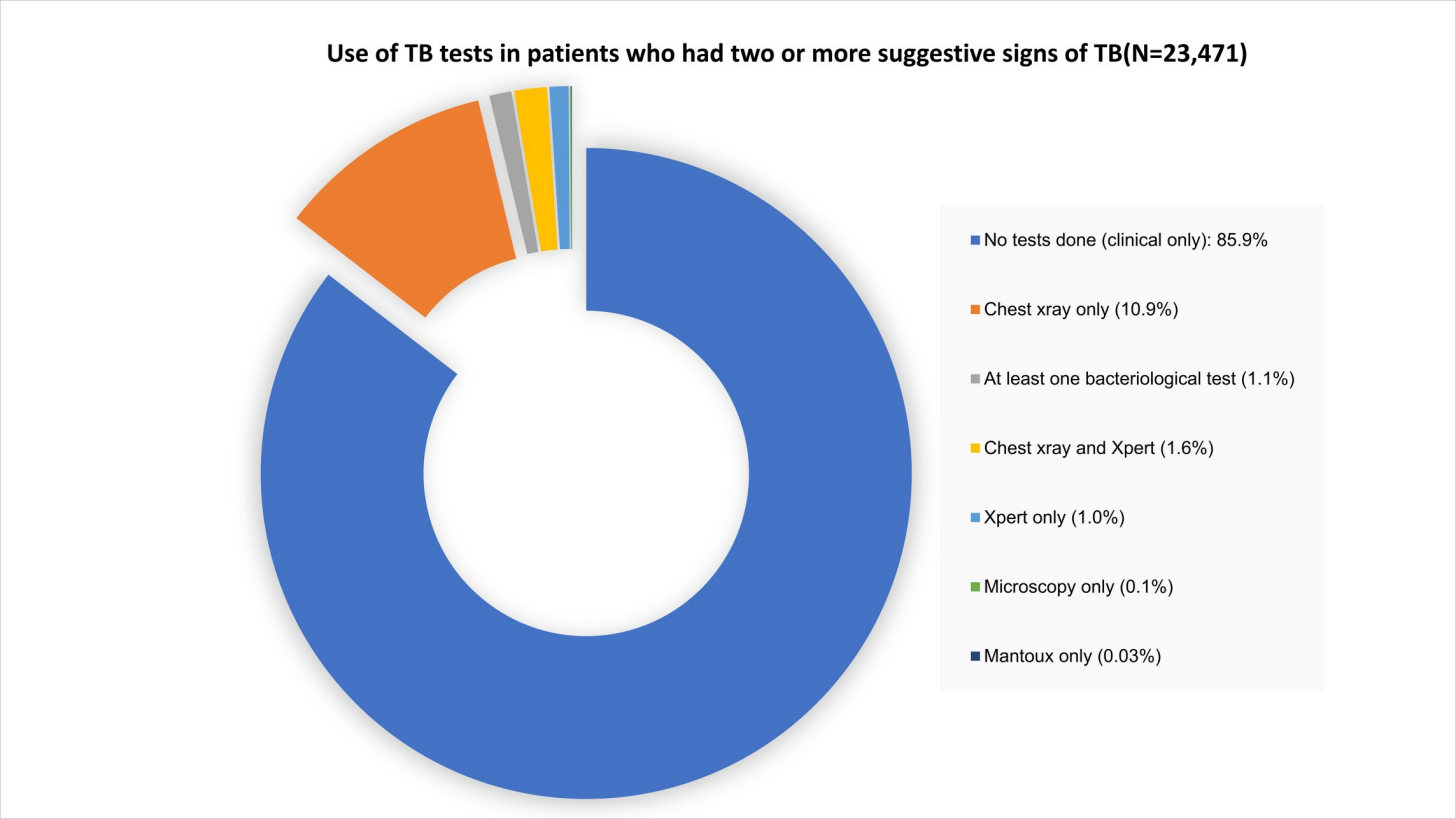
Use of TB tests in eligible patients.

We further explored use of TB tests, and from Fig 4, we found majority 20,392 (85.9%) of those who had two or more suggestive signs of TB (cascade step 1, n = 23,741) had no evidence of TB tests being requested. Chest X-ray was the most commonly requested TB test in 10.9% (2,576) of these patients, while 1.1% (261) had at least one bacteriological test requested with guideline recommended 1^st^ line Xpert/MTB RIF only requested in 1.0% (226) of these patients who met Step 1.

### Factors explaining TB test requests among patients who had two or more suggestive signs of TB (Step 1)

Table 3 shows associations between *a priori* specified variables and use of TB tests amongst patients admitted in paediatric wards in Kenya who had two or more suggestive signs of TB (step 1). In the unadjusted models (with hospital as random effect), patients who had greatest odds of having TB tests requested included: HIV infected/sero-exposed (OR 4.44, 95%CI 3.67 to 5.36); as those who had malnutrition (OR 2.72, 95%CI 2.49 to 2.97); severe pneumonia (OR 2.03, 95% CI 1.85 to 2.22); children >5yr (OR1.37, 95% CI 1.22 to 1.53); readmissions (OR 1.28, 95% CI 1.14 to 1.44); and referrals (OR 1.23, 95% CI 1.12 to 1.35). Male children had lower odds of getting TB tests compared to female patients (OR 0.90, 95% CI 0.83 to 0.97). Patients from hospitals in malaria endemic areas also had lower odds (OR 0.33, 95%CI 0.14 to 0.79).

**Table 3:** Factors associated with TB tests in patients having two or more suggestive signs of TB.

Characteristic	Full cohort who met Step 1 N = 23,741	Proportion who met Step 1 criteria with TB tests requested n = 3,350 (row %)	Unadjusted odds of getting TB tests requested (95% CI) N = 23,741	Adjusted odds of getting TB tests requested (95% CI) N = 18,282
**Patients admitted in each time period**				
*Period 1 (01*^*st*^ *Nov 15 to 31*^*st*^ *Oct 16)*	12,698	1,581 (47.2)	1 (ref)	1 (ref)
*Period 2 (01*^*st*^ *Nov 17 to 31*^*st*^ *Oct 18)*	10,773	1,769 (52.8)	1.22 (1.13 to 1.31)	1.19 (1.09 to 1.31)
**LEVEL 1: INDIVIDUAL LEVEL FACTORS**				
**Age**				
*Under 5yr*	20,319	2,851 (14.0)	1 (ref)	1 (ref)
*Over 5yr*	3,257	477 (14.7)	1.37 (1.22 to 1.53)	1.70 (1.55 to 2.05)
**Gender**				
*Female*	10,467	1,551 (14.8)	1 (ref)	1 (ref)
*Male*	13,202	1,787 (13.5)	0.90 (0.83 to 0.97)	0.91 (0.83 to 1.00)
**Readmission cases**				
*No*	17,404	2,413 (13.9)	1 (ref)	1 (ref)
*Yes*	2,463	471 (19.1)	1.28 (1.14 to 1.44)	1.24 (1.08 to 1.42)
**Referral cases**				
*No*	15,601	2,004 (12.8)	1 (ref)	1 (ref)
*Yes*	4,244	871 (20.5)	1.23 (1.12 to 1.35)	1.15 (1.04 to 1.28)
**Admission diagnosis of HIV**				
*Not HIV infected*	23,193	3,117 (13.4)	1 (ref)	1 (ref)
*HIV infected or exposed*	548	233 (42.5)	4.44 (3.67 to 5.36)	3.81 (3.05 to 4.75)
**Pneumonia with WHO guideline severity classification [37]**				
*No pneumonia (cold/URTI)*	10,633	973 (9.2)	1 (ref)	1 (ref)
*Pneumonia (non-severe)*	5,080	848 (16.7)	1.83 (1.65 to 2.03)	2.50 (2.14 to 2.91)
*Severe pneumonia*	8,028	1,529 (19.1)	2.03 (1.85 to 2.22)	2.98 (2.60 to 3.40)
**Admission diagnosis of malnutrition**				
*No malnutrition*	19,749	2,164 (11.0)	1 (ref)	1 (ref)
*Malnutrition*	3,992	1,187 (29.7)	2.72 (2.49 to 2.97)	2.98 (2.69 to 3.31)
**Effect of admission diagnosis of malnutrition interacting with pneumonia**				
*No malnutrition + no pneumonia*	19,748	2,163 (11.0)	1 (ref)	1 (ref)
*Malnutrition + no pneumonia*	1,869	445 (23.8)	3.92 (3.39 to 4.53)	4.67 (3.94 to 5.54)
*Malnutrition + non- severe pneumonia*	875	308 (35.2)	6.31 (5.30 to 7.50)	7.08 (5.76 to 8.70)
*Malnutrition + severe pneumonia*	1,249	434 (34.6)	5.95 (5.10 to 6.95)	6.37 (5.33 to 7.62)
**Presence of any danger sign**				
*No*	13,558	1,698 (12.5)	1 (ref)	
*Yes*	10,183	1,652 (16.2)	1.21 (1.12 to 1.31)	
**LEVEL 2: HOSPITAL LEVEL FACTORS (ICC 0.12, 95% CI 0.06 to 0.24)**				
**Patients from hospitals in malaria transmission areas**				
*Low malaria transmission*	14,933	2,705 (18.1)	1 (ref)	1 (ref)
*High malaria transmission*	8,808	645 (7.3)	0.33 (0.14 to 0.79)	0.41 (0.19 to 0.89)
**Patients from hospitals with high admissions (mean = 3239)**				
*Low admissions*	11,165	1,962 (17.6)	1 (ref)	
*High admissions*	12,576	1,388 (11.0)	0.89 (0.32 to 2.50)	
**Patients from hospitals in counties with high HIV prevalence**				
*Lower HIV prevalence*	15,512	2,160 (13.9)	1 (ref)	
*Higher HIV prevalence*	8,229	1,190 (14.5)	0.55 (0.19 to 1.62)	

We included 18,282 children in a complete case multivariable analysis and after adjusting for other variables and with hospitals as a random effect, patients diagnosed with malnutrition now had nearly five times the odds of getting TB tests requested compared to those without this diagnosis (AOR 4.68, 95% CI 3.95 to 5.54), with evidence of interaction between malnutrition and those who had pneumonia which increased the odds of getting TB tests. Effects of readmission, referral, having an HIV diagnosis and older age were all still associated with greater odds of getting TB tests in the adjusted model, while effect of gender was no longer statistically significant (Table 3). Patients from malaria endemic areas still had reduced odds of getting TB tests done. From the intraclass correlation coefficient (ICC), hospital as a level explained approximately 12% of the variability observed in the data.

### Estimated burden of TB amongst all paediatric admissions to Kenyan hospitals

Using our pre-specified case definitions, we provide a range of estimates for TB burden (Table 4). Clinicians documented a diagnosis of TB in 1,100/42,107 (2.6%) paediatric admissions, with highest reported in H5 (142/2,346, 6.1%) and lowest in H4 (16/1,954, 0.8%). This number increased by 102 patients to 1,202/42,107 (2.9%) when we included those who got anti-TB prescription but TB diagnosis was not documented. Only 234/42,107 (0.6%) of the admissions were documented to have met the guideline criteria for clinical TB diagnosis (met Steps 1–3 of the cascade), while only 89/42,107 (0.2%) patients from 13 hospitals over two calendar years had documented evidence of bacteriologically confirmed TB. Overall, 4,245/42,107 (10.1%) of all admissions had evidence of at least one TB test being requested.

**Table 4:** Hospital level characteristics and estimated burden of TB amongst admissions over 24 calendar months.

Hospital	Paediatric Admissions n (col%)	Clinician TB Diagnosis* n (row%)	Working TB Diagnosis* n (row%)	Clinical TB Diagnosis* n (row%)	Bacteriologically confirmed TB* n (row%)	TB tests requested* n (row%)
**H1**	3,429 (8.1)	34 (1.0)	35 (1.0)	8 (0.2)	0 (0)	187 (5.5)
**H2**	3,128 (7.4)	128 (4.1)	138 (4.4)	41 (1.3)	4 (0.1)	378 (12.1)
**H3**	4,154 (9.9)	70 (1.7)	72 (1.7)	11 (0.3)	2 (0.1)	284 (6.8)
**H4**	1,954 (4.6)	16 (0.8)	17 (0.9)	7 (0.4)	3 (0.2)	114 (5.8)
**H5**	2,346 (5.6)	142 (6.1)	155 (6.6)	17 (0.7)	2 (0.1)	352 (15.0)
**H6**	5,186 (12.3)	55 (1.1)	56 (1.1)	6 (0.1)	8 (0.2)	237 (4.6)
**H7**	3,135 (7.5)	33 (1.1)	37 (1.2)	6 (0.2)	4 (0.1)	21 (0.7)
**H8**	3,773 (9.0)	61 (1.6)	61 (1.6)	21 (0.6)	9 (0.2)	266 (7.1)
**H9**	2,479 (5.9)	124 (5.0)	139 (5.6)	33 (1.3)	37 (1.5)	368 (14.8)
**H10**	3,731 (8.9)	90 (2.4)	95 (2.6)	19 (0.5)	3 (0.1)	221 (5.9)
**H11**	3,180 (7.6)	172 (5.4)	183 (5.8)	45 (1.4)	16 (0.5)	1,008 (31.7)
**H12**	3,583 (8.5)	151 (4.2)	185 (5.2)	18 (0.5)	1 (0.03)	695 (19.4)
**H13**	2,029 (4.8)	24 (1.2)	29 (1.4)	2 (0.1)	0 (0)	115 (5.7)
**Total**	42,107	1,100 (2.6)	1,202 (2.9)	234 (0.6)	89 (0.2)	4,245 (10.1)

*N/B Denominator for columns is the total number paediatric admissions in each hospital for pooled time period

## Discussion

We explored TB diagnostic practices amongst paediatric admissions in Kenya using a conceptualised care cascade based on clinical guidelines, and found 56.4% of all admissions met step 1 of the cascade, with two or more signs and symptoms suggestive of TB. Step 2, further screening of history of TB contact or full respiratory exam was done in 62.6% who met Step 1. Step 3, a chest x-ray or Mantoux test was requested in 16.5% who met Step 2. Step 4, at least one bacteriological test was requested in 15.9% who met Step 3. In Step 5, a Working TB diagnosis was documented in 44.6% who met Step 4. Factors associated with requests of TB tests amongst patients who entered the cascade included: i) older age; ii) co-morbidities of HIV, malnutrition or pneumonia; iii) and severe disease, with sicker children, or those being readmitted or referred. The estimated burden of TB in children admitted to Kenyan hospitals by “Working TB Diagnosis” was 2.9%, and only 0.2% all admissions had bacteriologically confirmed TB.

If more than half of all admissions went on to get TB tests as implied by guidelines, this could potentially put a strain on stretched hospital resources due to time, cost and effort to test every eligible patient. We noted similar findings in an analysis of Kenya national TB programme data, with underuse of TB diagnostic tests both in children and adults and a preference for clinical diagnosis [8]. This has been observed in other high burden settings, which report the greatest gap in the TB care cascade being failure to get investigations [26, 38]. Clinicians appear to resort to their own judgement, selecting patients who are malnourished, HIV infected, referrals/readmissions, probably due to how non-specific guidelines seem to be.

TB symptoms of cough, fever, weight loss and lethargy mimic many other child hood diseases including malaria, pneumonia, malnutrition and HIV [39–41]. The investigations are also difficult to interpret in children who have pauci-bacillary disease, non-specific radiology findings and scanty sputum samples all contributing to reduced sensitivity and specificity of tests, which is compounded by lack of an appropriate gold-standard [15, 39]. Diagnostic criteria or scoring systems using constellations of clinical, radiological and laboratory findings are recommended by some to support clinical decision making [42]. The WHO criteria on which Kenyan guidelines are based, are widely in use and do not promote use of scoring systems [13, 43]. Our study however revealed that clinicians seem to prefer their own acumen, which begs the question: are guidelines helping? Clinicians’ preferences are probably influenced by other nuanced factors like co-morbidities or severity of illness, even in patients who met guideline criteria for further investigations.

Guidelines ideally should help improve effectiveness, reduce variations in clinical practice as well as mistakes and adverse events, and are key in quality of care [44]. Studies have shown even though TB diagnostic criteria and scoring systems have been widely used since the 1960’s, their reliability and validity remain unclear, especially in low resource settings where other co-morbidities like HIV, malnutrition and pneumonia are common [45, 46].This contributes to the difficulty of understanding the true burden of TB in children. We used a case definition of “Working TB Diagnosis” and found TB represented 2.9% of all admissions. If clinicians adhered strictly to the guidelines, there might be much more testing and probably more TB diagnoses documented. As such, we are still uncertain about the true burden of TB in admitted children in Kenya.

The proportion of tuberculosis amongst admitted children in high burden countries has scarcely been reported, and case definitions vary widely from setting to setting. In Papua New Guinea, they reported TB in 8% of admitted children, and this was based on a clinical score system [12, 47]. A Ugandan study found a TB occurrence of 18.9% in children admitted with pneumonia, using confirmed or probable case definitions [48]- this translated to a proportion of 1.1% amongst all admissions (51 TB cases/4,774 admissions). [49]. Our study found a slightly higher proportion than was noted in Uganda when we considered the “Working TB” diagnosis, probably because this definition included those who got anti-TB medication prescribed despite undocumented diagnosis. However, due to earlier reported issues of under-reporting and under-detection of TB in children in Kenya, and lack of adherence to guidelines, we believe our estimates are likely to be conservative, with a good number of cases still undetected [7, 8].

More work is required to continue to understand the complex epidemiology of TB in children, with a need for better surveillance/reliable data [9, 50, 51]. Understanding the epidemiology will guide clinicians especially in high burden settings like ours to develop a higher index of suspicion for TB. A review showed most TB patients are diagnosed after several weeks or months and multiple visits to health facilities[52]. Improved guidelines should provide clarity as to which patients should be investigated when, the order of doing investigations, including what to do in case tests are negative but one has a high index of suspicion for TB, as can be seen in the example of the Indian diagnostic algorithm [53]. A trade-off is needed between sensitivity and specificity, our data from clinician practice suggest that they weigh heavily on specificity and thus reject guideline recommendations.

A recent review suggested using existing tools and improving quality of care could potentially reduce TB deaths by half, but this all depends on having good clinical tools and investigations to identify cases that clinicians can rely on [54, 55]. We need to continuously review quality of care in our facilities, as several TB deaths occur despite patients seeking medical care [54]. This was seen in the Kenya national TB prevalence survey where three quarters of the patients went to health facilities with signs and symptoms suggestive of TB, but were never diagnosed [7]. Our results give the best estimates we have at present of the burden of TB in hospitalised children in Kenya and an audit of quality of care given to children with probable tuberculosis by using the cascade model to describe diagnostic practices, highlighting gaps in use of investigations that could be targeted in future quality improvement activities.

### Limitations

We observed some missing data, especially of laboratory results, and therefore could not infer if tests requested were done and what results were, despite efforts to trace back results. We did not impute missing data as missingness of clinical information is itself an aspect of the cascade itself, which we needed to note, as part of the audit of clinical care. There is also no unique identifier to link in-patient and out-patient records, which are often paper based, so we did not have access to follow-up post-discharge information for our study population. We however only included patients in whom a structured admission record form (PAR) was used, previous CIN analyses have shown more than 90% documentation in sites where these forms are used, so we are reasonably certain of documentation of test requests.

Data were collected around a time of civil strikes that caused disruptions in the health system and may have affected health seeking behaviour and health-worker morale. We dealt with this by taking calendar years before and after the strikes, and found admission characteristics were similar in the two study periods and therefore used pooled data for analysis. Our patient sample was large (more than 40,000 admissions), and from diverse sites. We are confident that we present the best available estimates of TB burden from routine paediatric inpatient data in Kenya as well as diagnostic practices [56].

Unlike other cascade studies, we did not have information on whether the patients completed TB treatment and what their outcomes were after, which would have required longer follow up of these patients. Our study how ever presents the first attempt in our knowledge to describe the burden of paediatric TB and diagnostic practices in a large group of in-patients over a two-year period, highlighting gaps that can aid in planning future interventions and studies as described by Ramnath Subbaramann et al, who describe potential uses of the TB care cascade [57].

### Conclusions: Generalisability/application

This work helps contribute to much-needed information on the burden of TB in children. With global efforts being harnessed to find the missing TB cases in the WHO END TB Strategy [58], patients already accessing health care are a potential low-hanging target for improving case detection. We need a better understanding of which children may have TB in our setting and how they present with clearer guidelines to help clinicians better select which patients to investigate, and how to interpret test results considering low sensitivity/specificity of available tests. A cascade approach was useful to visualise critical gaps so that interventions can be targeted to improve quality of care.

Further qualitative work is needed to understand reasons behind health workers’ diagnostic practices despite existing national guidelines and availability of diagnostic tests in these facilities. Economic analyses could also shed more light onto resource implications of implementing the guidelines that recommend investigations for potentially more than half the admissions who had suggestive signs and symptoms of TB. More sensitive criteria would be helpful to discriminate those patients who would benefit from investigations including positive history of contact with a suspected/known TB patient or chronicity of cough both of which were poorly documented in our data. Clinicians therefore need additional support to improve their index of suspicion of TB amongst the admissions they see, in a high burden TB country like ours.

## Supporting information

S1 Chart Paediatric Admission Record (PAR). (PDF)

S1 Fig Excerpt from the Kenya Paediatric TB Guidelines. (TIF)

S2 Fig Patient Flow according to the cascade of care. (TIF)

S1 Table Definitions of key variables of interest. (DOCX)

S2 Table Absolute patient numbers in the cascade of care. (DOCX)
