# Supplementary material for: Diagnostic practices and estimated burden of tuberculosis among children admitted to 13 government hospitals in Kenya: An analysis of two years’ routine clinical data
Source: PLoS One. 2019 Sep 4;14(9):e0221145. doi: 10.1371/journal.pone.0221145 (PMC6726144; doi:10.1371/journal.pone.0221145)
Supplement: S1 Chart — (PDF) [file pone.0221145.s001.pdf]

Examination

Vital Signs	Temp	°C	Resp Rate	bpm	HR	/min	O2 Sat	%	BP	mmHg

General Examination

Oral thrush Y☐ N☐ Lymph N > 1cm Y☐ N☐

Finger Clubbing Y☐ N☐

Jaundice		0	+	+++
Oedema		☐None ☐Foot	☐Knee	☐Face
A	Stridor		Y☐	N☐
B	Central Cyanosis		Y☐	N☐
	Indrawing		Y☐	N☐
	Grunting		Y☐	N☐
	Acidotic breathing		Y☐	N☐
	Wheeze		Y☐	N☐
	Crackles		Y☐	N☐
Circ & Dehy dr'n	Peripheral Pulse	☐Normal	☐ Weak	
	Cap Refill	secs	*X = not possible*	
	Pallor / Anaemia	0	+	+++
	Skin warm at:	☐ Hand	☐ Elbow	☐ Shoulder
	Sunken eyes		Y☐	N☐
	Skin pinch (sec)	0	1	≥ 2
D	AVPU	A	V	P	U
	Can drink / breastfeed?		Y☐	N☐
	Stiff neck		Y☐	N☐
	Bulging fontanelle		Y☐	N☐
Infant < 2m	Irritable		Y☐	N☐
	Reduced movement / tone		Y☐	N☐

Abdomen

Rt Lt

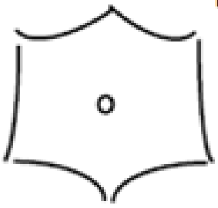

Chest

R L

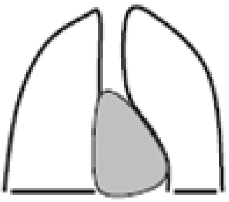

Front

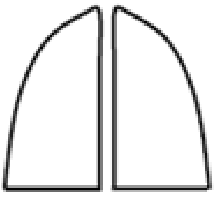

Back

CVS

Bones & Joints

Wrist / Rib signs Rickets Y☐ N☐

ENT exam	Neurological Examination
Rt Ear<br><br>Lt Ear<br><br>Nose<br><br>Throat	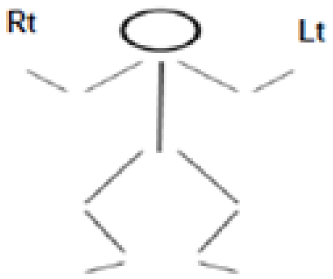

Investigations Ordered (record subsequent tests and all results in medical record)

Malaria	☐ Blood slide ☐ Rapid Test	*Glucose*	☐ Stick test ☐ Laboratory
Haematology	☐ Hb ☐ HCT ☐ Full haemogram	*Chemistry*	☐ Na + K ☐ U&C ☐ Ca ☐ Alb ☐ LFT
Microbiology	☐ Lumbar Puncture ☐ Blood Culture	*HIV*	☐ Rapid test ☐ PCR
X-Ray	☐ CXR ☐Wrist Other =	*Urine*	☐ Urinalysis ☐ Micro & culture
TB Test	☐ Microscopy for AAFBs<br>☐ Xpert MTB/RIF ☐ *Myco. TB* culture	*Other 1*	
		Other 2	

Summary of presentation & problems

Admission Diagnoses – Select ONE *primary diagnosis (tick box indicating "1") and ANY secondary diagnoses (tick box indicating "2"), then indicate level of severity or type of disease if required*

Malaria	1 ☐ 2 ☐	☐ Severe ☐ Non-severe	*Anaemia*	1 ☐ 2 ☐	☐ Severe ☐ Non-severe	
Pneumonia	1 ☐ 2 ☐	☐ Severe ☐ Non-severe	*Sickle cell disease*			1 ☐ 2 ☐
Diarrhoea	1 ☐ 2 ☐	☐ Non-bloody ☐ Bloody (dysentery)	*Meningitis*			1 ☐ 2 ☐
Dehydration	1 ☐ 2 ☐	☐ Shock ☐ Severe ☐ Some	*Rickets*			1 ☐ 2 ☐
HIV	1 ☐ 2 ☐	☐ Positive ☐ Exposed /PMTCT +<br>☐ Negative ☐ Declined test	*Asthma*	1☐ 2☐	☐ Severe<br>☐ Mild/moderate	
Malnutrition	1 ☐ 2 ☐	☐ Kwash ☐ Marasm ☐ M. Kwash<br>☐ Moderate malnutrition	*Suspected TB*			1 ☐ 2 ☐
			Prematurity / LBW			1 ☐ 2 ☐
Other 1	1 ☐ 2 ☐		*Neonatal sepsis*			1 ☐ 2 ☐
Other 2	1 ☐ 2 ☐					

Treatment Supportive care & Observations – indicate what care is needed

Keep warm	☐	Oxygen	☐	**Clinician Name & Sign**
IV / oral fluids plan	☐	Blood transfusion	☐	……………… ………………
Vitamin A	☐	Nutrition / Feeds plan	☐	
Review status	☐ Medical <6hrs ☐ Priority Nursing Observations			Date… /……/…… Time …………<br>dd/mm/yyyy am / pm
