# Supplementary figures and images for: Diagnostic practices and estimated burden of tuberculosis among children admitted to 13 government hospitals in Kenya: An analysis of two years’ routine clinical data
Source: PLoS One. 2019 Sep 4;14(9):e0221145. doi: 10.1371/journal.pone.0221145 (PMC6726144; doi:10.1371/journal.pone.0221145)

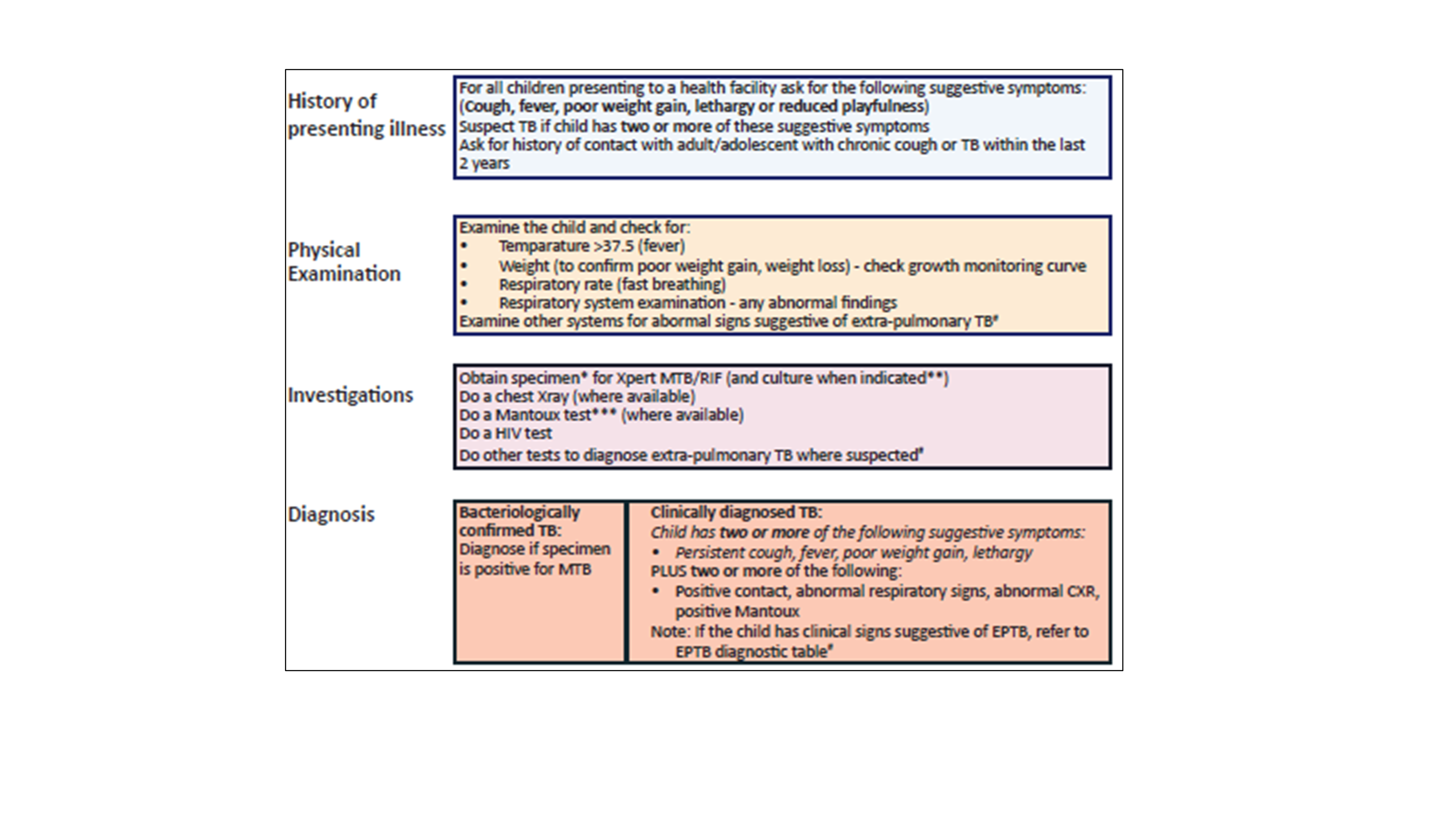

Supplement: S1 Fig — (TIF) [file pone.0221145.s002.tif]

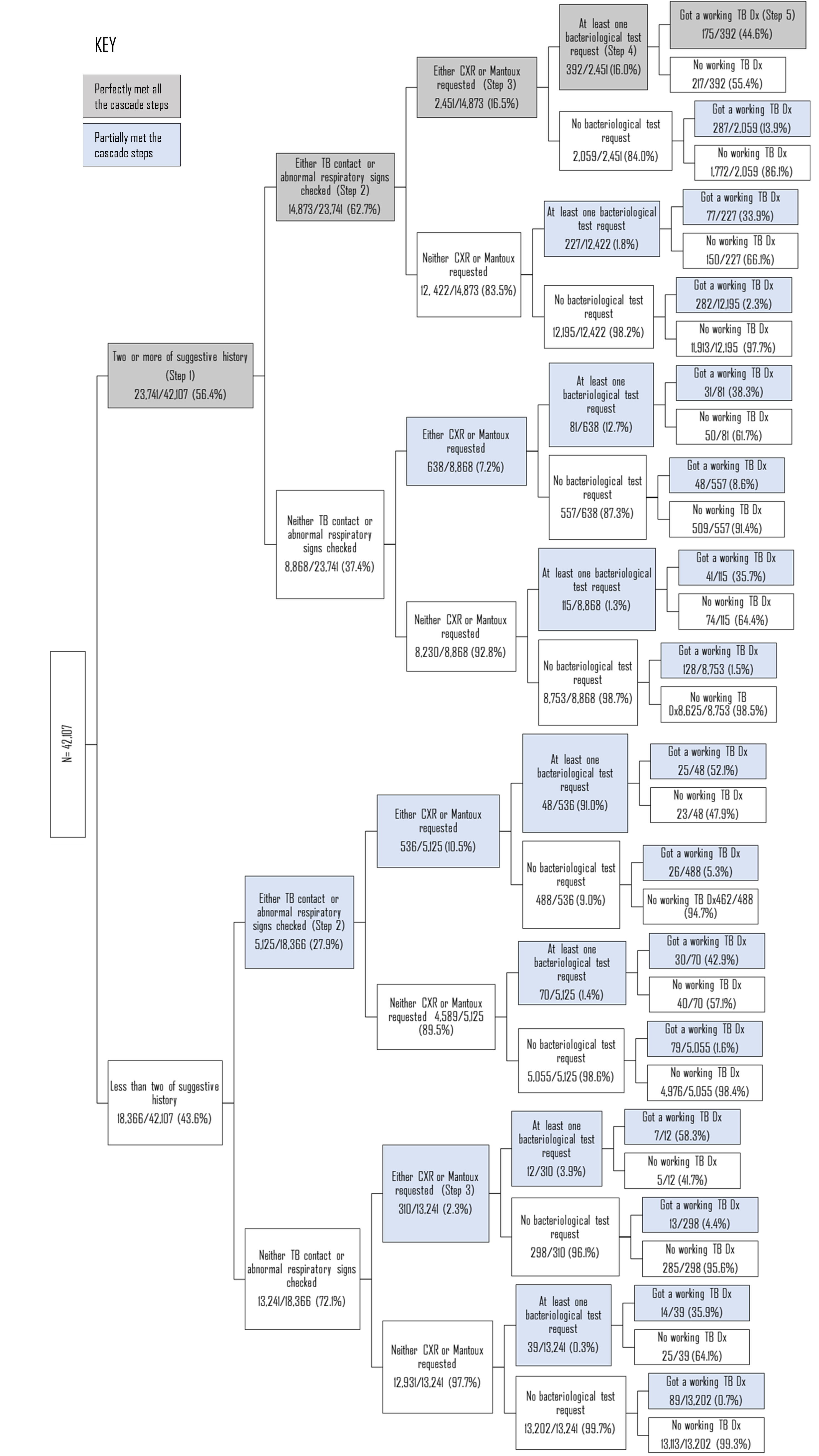

Supplement: S2 Fig — (TIF) [file pone.0221145.s003.tif]
