# Supplementary material for: Diagnostic practices and estimated burden of tuberculosis among children admitted to 13 government hospitals in Kenya: An analysis of two years’ routine clinical data
Source: PLoS One. 2019 Sep 4;14(9):e0221145. doi: 10.1371/journal.pone.0221145 (PMC6726144; doi:10.1371/journal.pone.0221145)
Supplement: S1 Table — (DOCX) [file pone.0221145.s004.docx]

S3 Table of definitions of key variables of interest

| Variable | Definition |
| --- | --- |
| Underweight | *< -2SD Weight for age Z score (using CDC charts for 0-20yrs)* |
| Growth faltering | *Either WAZ <-2SD* **OR** *admission diagnosis of malnutrition/failure to thrive* **OR** *had a prescription for supplementary feeds* |
| Lethargic | *AVPU < Alert* **OR** *not able to drink* |
| Danger signs | *Central cyanosis* **OR** *AVPU < Alert* **OR** *not able to drink* **OR** *grunting* **OR** *received oxygen* |
| Abnormal respiratory sign | *High respiratory rate (for age)* **OR** *received oxygen* **OR** *central cyanosis* **OR** *indrawing* **OR** *grunting* **OR** *acidotic breathing* **OR** *crackles* **OR** *wheeze* |
| Severe chest signs (as per Kenya Guidelines) | *Oxygen saturation <90%* **OR** *central cyanosis* **OR** *not able to drink* **OR** *AVPU < Alert* **OR** *grunting* |
| Severe pneumonia | *Had cough/difficulty breathing* **AND** *at least one of the severe signs* |
| Non-severe chest signs | *Indrawing* **OR** *high respiratory rate for age* |
| Non-severe pneumonia | *Had cough/difficulty breathing* **AND** *none of the severe signs* **AND** *at least one of the none severe signs* |
