# Supplementary material for: Diagnostic practices and estimated burden of tuberculosis among children admitted to 13 government hospitals in Kenya: An analysis of two years’ routine clinical data
Source: PLoS One. 2019 Sep 4;14(9):e0221145. doi: 10.1371/journal.pone.0221145 (PMC6726144; doi:10.1371/journal.pone.0221145)
Supplement: S2 Table — (DOCX) [file pone.0221145.s005.docx]

S4 Patients who met criteria for the paediatric TB cascade of care steps

|  | N= 42,107 |
| --- | --- |
| Step 1. Two or more suggestive signs and symptoms (cough, fever, lethargy, growth faltering) | 23,741 (100) |
| Step 2. Further screening done for TB risk done in those who met step 1 (checked for positive TB contact or abnormal respiratory signs) | 14,873/23,741 (62.6) |
| Step 3: Initial investigations to support clinical TB diagnosis requested for those who met step 1 and 2 (chest x-ray or Mantoux test requested) | 2,451/23,741 (10.3) |
| Step 4. Patients who met steps 1-3 and had at least one bacteriological investigation requested (Xpert® or microscopy or culture) | 392/23,741 (1.7) |
| Step 5. Patients who met steps 1-4 and got a Working TB diagnosis | 175/23,741 (0.7) |
